# The Phylogeographic History of the New World Screwworm Fly, Inferred by Approximate Bayesian Computation Analysis

**DOI:** 10.1371/journal.pone.0076168

**Published:** 2013-10-02

**Authors:** Pablo Fresia, Ana Maria L. Azeredo-Espin, Mariana L. Lyra

**Affiliations:** 1 Departamento de Entomologia e Acarologia, Escola Superior de Agricultura "Luiz de Queiroz", Universidade de São Paulo, São Paulo, Brasil; 2 Centro de Biologia Molecular e Engenharia Genética and Instituto de Biologia, Universidade Estadual de Campinas, São Paulo, Brasil; 3 Departamento de Zoologia, Instituto de Biociências, Universidade Estadual Paulista, São Paulo, Brasil; George Washington University, United States of America

## Abstract

Insect pest phylogeography might be shaped both by biogeographic events and by human influence. Here, we conducted an approximate Bayesian computation (ABC) analysis to investigate the phylogeography of the New World screwworm fly, *Cochliomyia hominivorax*, with the aim of understanding its population history and its order and time of divergence. Our ABC analysis supports that populations spread from North to South in the Americas, in at least two different moments. The first split occurred between the North/Central American and South American populations in the end of the Last Glacial Maximum (15,300-19,000 YBP). The second split occurred between the North and South Amazonian populations in the transition between the Pleistocene and the Holocene eras (9,100-11,000 YBP). The species also experienced population expansion. Phylogenetic analysis likewise suggests this north to south colonization and Maxent models suggest an increase in the number of suitable areas in South America from the past to present. We found that the phylogeographic patterns observed in *C. hominivorax* cannot be explained only by climatic oscillations and can be connected to host population histories. Interestingly we found these patterns are very coincident with general patterns of ancient human movements in the Americas, suggesting that humans might have played a crucial role in shaping the distribution and population structure of this insect pest. This work presents the first hypothesis test regarding the processes that shaped the current phylogeographic structure of *C. hominivorax* and represents an alternate perspective on investigating the problem of insect pests.

## Introduction

Phylogeography is a growing field that was originally conceived to explore the processes underlying geographic distribution of genetic diversity within and among species [1,2]. Biogeographic events such as climate oscillations and distribution of suitable habitats over time have been highlighted as key factors underlying the geographical variation within species [3-7]. In the Neotropics, many hypotheses have been proposed to explain how these biogeographic events might have shaped the current population structure of various taxa [8-12]. In particular, Haffer [13] and Vanzolini and Williams [14] proposed that the retraction of the Amazon rainforest and the accompanying fauna in different refuges during the Pleistocene glacial cycles might have promoted geographical speciation events.

Understanding the impacts of climate-induced distributional shifts on species divergence, such as those accompanying the Pleistocene glacial cycles, requires tools that explicitly incorporate models of past geographic distributions into analyses of genetic differentiation [15]. These tools are emerging from the recent integration of statistical genetics and geospatial methods with phylogeography [16,17]. These new techniques help place demographic events in a historical and spatial context and highlight some of the potential mechanisms underlying geographic distribution and diversification [17]. The use of this integrative approach may be especially helpful in clarifying the demographic history of species with complex histories of population expansion, gene flow and vicariance.

The distribution and demography of insect pest species are influenced not only by biogeographic events but also by human activities because insects attack livestock and/or crops. Increases in human and domestic animal populations and crops, as well as the worldwide movements of humans and their goods, have greatly accelerated the breakdown of barriers to species movements and insect population expansion [18]. Key factors in pest management strategies include a good knowledge of species’ phylogeographic structure and the ability to distinguish between the historical biogeographic events and contemporary human influences that shape the current geographical distribution of insect pests [19]. These factors provide insight into the current patterns of gene flow and can help determine the appropriate geographic scale for effective treatments [20-22]. Although it is challenging to separate these contemporary and historical effects in insect species demography, an integrative phylogeographic analysis, including explicit hypothesis tests, may provide a means to distinguish spatial and temporal factors.

*Cochliomyia hominivorax* Coquerel, 1858 (Diptera: Calliphoridae), the New World screwworm, is an endemic fly of the Americas [23]. The larval stages of this insect feed on the living tissue of warm-blooded hosts, including humans. Larval infestation causes serious tissues injuries and can lead to host death if not treated, and for that reason, *C. hominivorax* is considered an important livestock pest that causes substantial profit losses to livestock breeders [24]. The current distribution of *C. hominivorax* includes South America and some Caribbean islands; it was eradicated from North and continental Central America between 1957 and 2000 [25].

The association of this species with vertebrate hosts and its status as an insect pest suggest that the genetic and geographic distribution of this species is influenced by human activities. The genetic structure of *C. hominivorax* populations has already been analyzed on different geographical scales [26-32]. Two phylogeographic studies of *C. hominivorax* across its current distribution [31,32] revealed the continental-scale geographic structure of the populations and identified four regional groups (Cuba, Dominican Republic, and the North and South Amazon regions). At the intra-group level, the authors did not find that the population structure was associated with geography. They suggested that the human and livestock mobility in the continent during the last ~500 years might have obscured the historical phylogeographic pattern of *C. hominivorax* on this smaller scale but not on the continental scale [32].

Here, we focus on the mode and time of divergence of the regional groups of *C. hominivorax* on the continental scale. We used mitochondrial DNA sequences to generate phylogenetic reconstructions and habitat suitability models to construct alternative hypotheses of population divergence. An approximate Bayesian computation (ABC) approach was used to test these competing demographic history scenarios for *C. hominivorax* to identify contemporary and historical events that shaped the current population structure of this insect pest.

## Materials and Methods

### Samples and sequence data

We analyzed genetic data from 402 *C. hominivorax* individuals from 60 locations in South, Central and North America (Table S1). Of these, 361 individuals were obtained from our previous work [32], 26 were obtained from 15 new locations of South America, and the remaining 15 were obtained from five laboratory strains from North and Central, American locations sampled before the eradication program [25] and maintained by the APHIS-USDA Biofactory, Fargo, North Dakota, USA (Table S1). These laboratory samples represent the native populations from North America and continental Central America. In addition to these samples, we sequenced two individuals of *Cochliomyia macellaria* Fabricius 1775, one of the other three species of the genus, to be used as out-group in the phylogenetic analyses.

We analyzed 510 bp of the mitochondrial DNA (mtDNA) control region (CR), 731 bp of the mtDNA gene Cytochrome *c* oxidase subunit I (COI), and 511 bp of the mtDNA gene Cytochrome *c* oxidase subunit II (COII). The sampling details and laboratory protocols are described in Fresia et al. [32]. The 41 new samples were allocated within the regional groups previously described in Fresia et al. [32]. The genetic variability within groups and pairwise Φ_ST_ among groups were estimated using Arlequin software v3.11 [33].

### Phylogenetic analysis and demographic history

Nucleotide sequence alignment was performed with MEGA software v5.0 [34] using the ClustalW algorithm and the data from the three mtDNA fragments (CR, COI and COII) were combined in a 1752 bp fragment. The analyses were performed using this combined dataset. Unambiguous haplotypes selected with Fabox [35] were used for the subsequent phylogenetic analysis, and the *C. macellaria* sequences were used to root the in-group taxa.

The Akaike information criterion implemented in jModeltest software v0.1.1 [36] was used to select the best fitting model for each mtDNA fragment. The following models were selected: HKY+Γ for CR, HKY+I for COI and HKY+I+Γ for COII. A Bayesian phylogenetic inference was conducted with BEAST software v1.6.2 [37] using partitioned models to incorporate evolutionary information for each gene fragment. We unlinked the nucleotide substitution models for each gene partition and codon position (for COI and COII) and used an uncorrelated lognormal relaxed clock. We used the coalescent constant size model starting with a randomly generated tree. The chains ran for 80 million generations, and the tree parameters were sampled every 8000 generations; 10% of the initial values were discarded as burn-in. The convergence of the runs was confirmed using Tracer software [38], and the tree was summarized with TreeAnotator v1.6.2 using the maximum clade credibility option as the target tree type and mean heights for the node heights.

Bayesian Skyline plot (BSP) analyses, implemented in BEAST software v1.6.2 [37], were used to depict past demographic changes in *C. hominivorax* populations. Analyses were conducted for each phylogenetic main clade (see tree topology results) and also for regional groups within clades. Following Fresia et al. [32], we used just COI sequences and applied a strict molecular clock with substitution rate of 1% per million years per lineage. All BSP analyses consisted of either 50 or 80 million MCMC iterations with the first 25% discarded as burn-in, and parameters sampled every five or eight thousand steps.

### Habitat suitability modeling

Various methods can be used for species habitat suitability modeling from presence-only data and digital environmental maps [39,40]. This approach uses geographic information system methods (GIS) to extract the environmental data associated with geo-referenced sampling locations, which are projected into the geographic space to generate a model of species habitat suitability. The resulting model can then be used to generate a map of the predicted species potential distribution based on the climate variables for the past, present and/or future [41].

We used Maxent software v3.3.3k [42] to compute habitat suitability models (HSMs) for *C. hominivorax*. The Maxent algorithm estimates the habitat suitability of a species from environmental data on occurrence locations and finds the maximum entropy distribution to predict where the species may occur based on the environmental similarities with the sampled locations [42]. We obtained 121 species records from our own fieldwork and from the literature (Table S2), including data recorded prior to the eradication program (see 25) from North and Central America. The climate variables in the current data, with a 2.5 arcmin resolution (~5 Km^2^), were downloaded from the WorldClim v1.4 database [43] (http://www.worldclim.org) along with simulations describing the conditions during the Last Glacial Maximum (LGM ~21,000 years BP; using MIROC and CCSM models) and the last interglacial era (LIG; ~120,000-140,000 years BP). A mask shape file that includes the Americas without Canada and Alaska was used to clip the climate data with the GDAL/OGR Library (http://gdal.org). Correlations between the 19 BIOCLIM variables [44] were estimated using ENMTools software v1.3 [45,46]. The non-correlated variables (*R* ^2^<0.7) for the computed models included the following: mean monthly temperature range (BIO2), maximum temperature of the warmest month (BIO5), minimum temperature of the coldest month (BIO6), mean temperature of the wettest quarter (BIO8), precipitation seasonality (BIO15), precipitation of the warmest quarter (BIO18) and precipitation of the coldest quarter (BIO19).

Ensemble model predictions may produce more reliable and robust HSM results [47]; therefore, we used the cross-validation option with 10 replicates. These models were evaluated via AUC statistics (area under the receiver operating characteristic curve) [48]. For the threshold between the suitable and unsuitable conditions, we applied a cumulative probability of 10, which rejects 10% of the presence observations (omission of 10%). Although arbitrary, this level was selected to provide a more conservative interpretation of habitat suitability because it still has a low omission rate [49]. The average between CCSM and MIROC models was used to evaluate the suitability regions on the LGM.

### Approximate Bayesian computation analysis

Statistical phylogeography is a hypothesis-testing framework that allows for formal tests of certainty as well as tests between competing models [50-52]. Within this framework, ABC coupled with coalescent modeling [53] is becoming the standard method to address population genetics and phylogeographical questions [54-56].

We used DIYABC software v1.0.4.39 [57] to compare competing hypotheses regarding demography and population divergence in *C. hominivorax* on the continental scale. The hypotheses were constructed primarily to test the order (from north to south or vice versa) and time of divergence of the population groups, as well as the possibility of population admixture after divergence. Our hypotheses were based on genetic diversity analysis, phylogenetic inference and habitat suitability models (see results). Because the phylogenetic analysis corroborated previous results [32] pertaining to colonization on the Caribbean islands, we focused on testing the relationships among mainland samples.

For the ABC simulations, we used the combined sequence dataset (three mtDNA fragments) and considered each regional group to be a “population”. The populations were as follows: (1) SAG (all samples from the South Amazon region, N = 249), (2) NAG (samples from the North Amazon region, excluding samples from Jamaica and Trinidad and Tobago, N = 53), and (3) NC (excluding samples from Cuba, N = 15). We also excluded three sequences from the NAG group that are considered to be human-mediated migrants (see 32) because the current version of DIYABC does not accommodate populations that exchange migrants [58].

**Figure 1:**
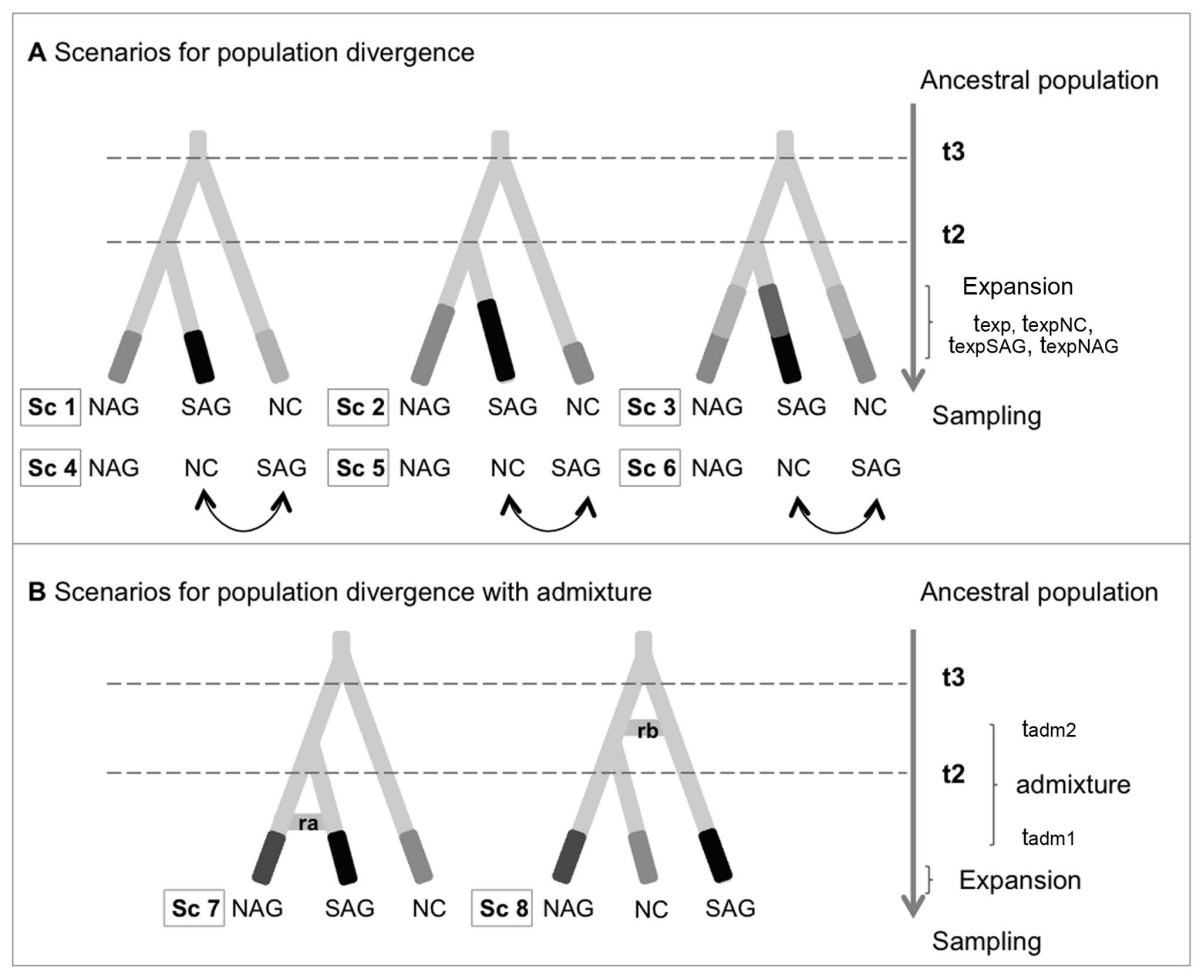
Competing scenarios of *C. hominivorax* regional group divergence and admixture. A: Sc1-Sc6: Divergence without admixture scenarios. B: Sc7-Sc8: NAG and SAG population admixture scenarios. NC: North and Central America group. NAG: North Amazon region group. SAG: South Amazon region group. t_2_ and t_3_: divergences times. ra, rb and rc: rates of admixture. t_exp_: expansion times. The different colors and widths of lines represent the effective sizes of different populations.

The hypothesis tests were carried out hierarchically. First, we compared six competing scenarios of population divergence without admixture (Figure 1A; Sc1, Sc2, Sc3, Sc4, Sc5 and Sc6). Then, we compared two competing scenarios of population admixture (Figure 1B; Sc7 and Sc8), and finally, we compared the best scenarios for each of the previous analyses. The population divergence scenarios differed in the order of population divergence and in the number and time of demographic expansion events. The population admixture scenarios considered admixture between the NAG and SAG groups at different times (Sc7, Sc8), i.e. before or after NC divergence.

Table 1 shows the prior definitions and distributions for all of the hypothetical scenarios. Before the final analyses, we run several trials to adjust the prior distributions in order to accommodate a wide range of effective population sizes and divergence times, even allowing equal divergence time for the tree regional groups. In all of the scenarios, the time of the first population split was set as t_3_ and the subsequent split was set as t_2_. We allowed the current populations to expand in one or more moments (t_exp,_ t_expNAG,_ t_expSAG,_ t_expNC_), and we set the conditions as t_exp_ ≤ t_2_ < t_3_. In the admixture scenarios, the admixture event time was set as t_adm1_ for Sc7 and ta_dm2_ for Sc8, where t_adm1_ < t_2_ and t_adm2_ > t_2_. Split times were translated into years by assuming a generation time (T) of 23.95 days, calculated from the formula T = α + [*s*/(1 -*s*)] [59], where α is the age of maturity and *s* is the survival rate of adults, which was estimated as 0.798 for *C. hominivorax* [60]. The population effective sizes were set as N_anc_ < N_NC_ ≤ N_NAG_ ≤ N_SAG_, and N1 to N8 > N_anc_. The parameters were simulated using a HKY model with 92% of the invariant sites and a gamma shape of 2.

**Table 1:** Definition and prior distribution of parameters used in the ABC tests of divergence scenarios.

		Distribution	
Parameters	Parameter name	Type	Interval
Effective sizes of the ancestral population	N_A_	Uniform	[1000-60000]
South Amazon population	N_SAG_	Uniform	[100000-3600000]
North Amazon population	N_NAG_	Uniform	[150000-800000]
North and Central America	N_NC_	Uniform	[40000-150000]
Population effective sizes between t_3_ and t_exp_	N1-N8	Uniform	[100-200000]
Expansion event	t_exp,_ t_expNAG,_ t_expSAG,_t_expNC_	Uniform	[60000-450000]
Admixture event	t_adm1,_ t_adm2_	Uniform	[20000-300000]
Time of the last population divergence	t_2_	Uniform	[50000-350000]
Time of the ancestral divergence	t_3_	Uniform	[100000-1000000]
Mean mutation rate (site/generation)	Mµ	Uniform	[10^-7^ -10^-11^]
Rate of admixture	ra, rb, rc	Uniform	[0.01–0.99]

The genetic variation within and among the three population samples was summarized based on the number of haplotypes, number of segregating sites, mean number of pairwise differences and all pairwise F_ST_’ s, totaling 12 summary statistics. We simulated one million data sets for each of the eight scenarios, and four million data sets for the comparison between the two best scenarios (~ two million each).

Following Cornuet et al. [58], we performed a logistic regression to estimate the (relative) posterior probability of each scenario, taking a number of simulated data sets closest to our real data set between 0.1% and 1%. The 95% credibility intervals for the posterior probabilities were computed through the limiting distribution of the maximum likelihood estimators. Once the most likely scenarios were assessed, we used a local linear regression to estimate the posterior distributions of parameters under this scenario. We chose the 1% of simulated data sets closest to our real data set for the logistic regression after applying a logit transformation to the parameter values. In order to evaluate the goodness-of-fit of the estimation procedure, we performed a model checking computation [57] by generating 1,000 pseudo-observed data sets with parameters values drawn from the posterior distribution given the most likely scenario.

## Results

### Genetic diversity

The combined mtDNA dataset (1752 bp) including the new and published sequences of *C. hominivorax* [32] has 230 haplotypes for the 402 individuals analyzed (Table S1 shows sample locations and haplotype distribution for each population; Table S3 shows the haplotype definition and GenBank accession numbers). Of the total number of haplotypes, 16 are new and were identified in the 41 individuals sequenced in the present work. These individuals were compared to all of the other samples and assigned to the four regional groups previously described in Fresia et al. [32], i.e., the Cuban group (CG), Dominican Republic group (DRG), northern Amazon group (NAG) and southern Amazon group (SAG). Although we did not find any shared haplotypes between the groups, we did find one (out of 230) shared between Cuba and Venezuela.

**Figure 2:**
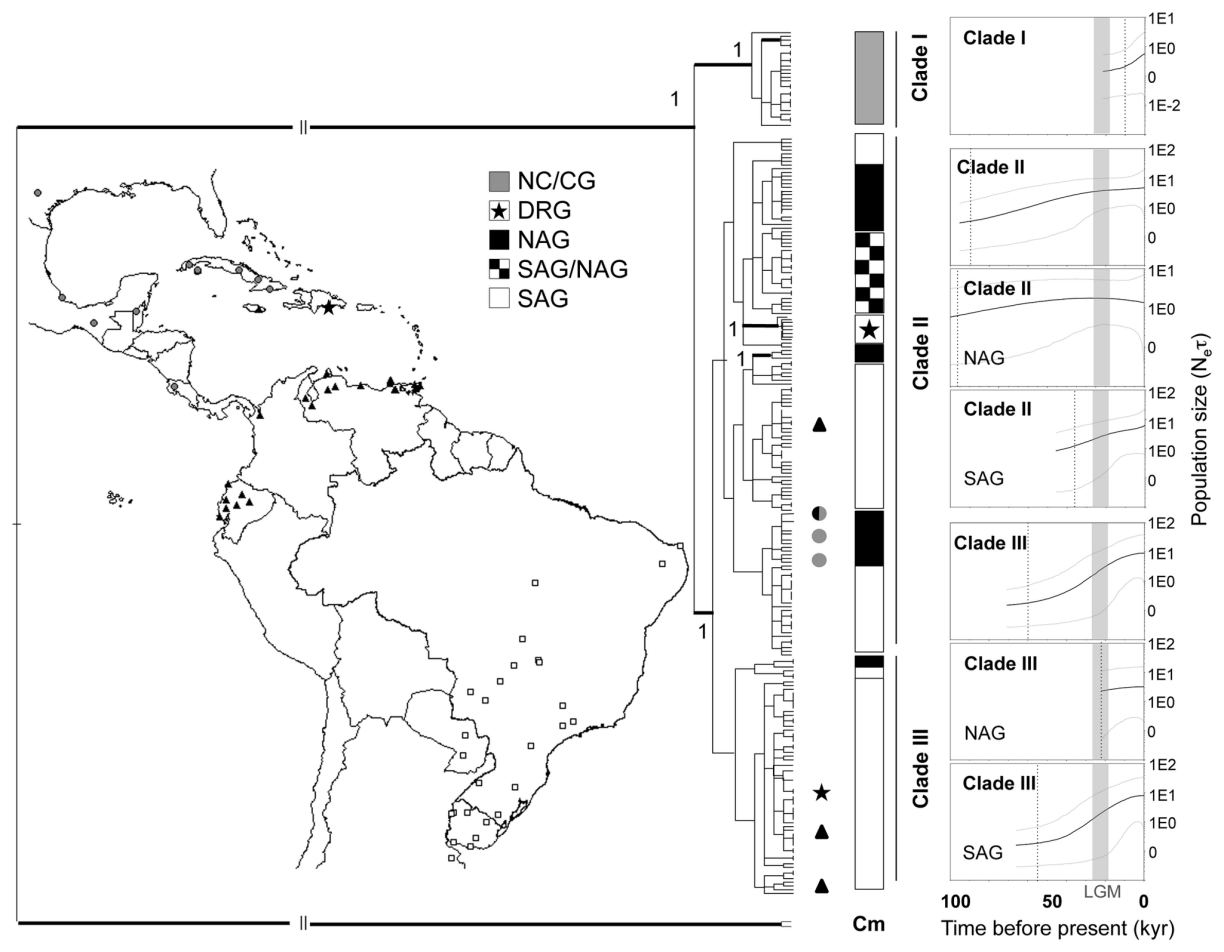
Sampled locations, Bayesian tree of haplotypes and Bayesian Skyline Plots. NAG: North Amazon regional group (black); SAG: South Amazon regional group (white); DRG: Dominican Republic regional group (black star); NC/CG: North American, Central American and Cuban Group (gray). Color bars besides tree shows haplotypes of each regional group. The Bayesian Skyline Plots (BSP) x-axis represents time in thousand years and y-axis the relative female population size. The dark solid line is the mean estimate, and the grey lines show the 95% highest posterior density limits. Gray bars in BSP graphs corresponds to the Last Glacial Maximum (~19-26,000 yr ago). Dotted lines show time that expansion began in each plot.

In the 15 North and Central American individuals, we identified six new haplotypes, which are closely related but not identical to the samples collected in Cuba and are genetically distant from the samples of South America and the other Caribbean islands (Figure 2). The group that includes these samples is referred to here as NC/CG and is equivalent to CG in Fresia et al. [32].

Table 2 shows the genetic variability of each regional group, and Table 3 shows the pairwise Φ_ST_ among groups. The SAG, NAG and NC/CG showed very similar haplotype and nucleotide diversities, and the DRG showed the smallest values for both indices. The pairwise Φ_ST_ estimates among groups support the previously observed genetic structure of *C. hominivorax* populations [32]. The high haplotype diversity (Ĥ) values and low π values for the entire dataset and regional subsets support a model of population and range expansion for *C. hominivorax* (see 61), as previously described in Fresia et al. [32].

**Table 2:** Genetic diversity estimates for *C. hominivorax* regional groups.

Regional group	N	N° hap	Ḧ ± SD	π ± SD
SAG	249	153	0.967 ± 0.008	0.0045 ± 0.0023
NAG	79	42	0.968 ± 0.009	0.0044 ± 0.0023
DRG	13	7	0.795 ± 0.109	0.0016 ± 0.0010
NC/CG	61	29	0.954 ± 0.011	0.0036 ± 0.0019
Total	402	230	0.985 ± 0.003	0.0063 ± 0.0031

NAG: North Amazon regional group; SAG: South Amazon regional group; DRG: Dominican Republic regional group; NC/CG: North and Central American and Cuban Group. N: number of individuals analyzed.

N° hap: Number of haplotypes, Ĥ: Haplotype diversity, π: Nucleotide diversity and SD: standard deviation.

**Table 3:** Pairwise Φ_ST_ among the four regional groups of *C. hominivorax*.

	SAG	NAG	DRG
NAG	0.155*		
DRG	0.347*	0.359*	
NC/CG	0.597*	0.623*	0.718*

NAG: North Amazon regional group; SAG: South Amazon regional group; DRG: Dominican Republic regional group; NC/CG: North American, Central American and Cuban Group. *P < 0.001

### Tree topology

The Bayesian phylogenetic reconstruction returned three main clades with a high posterior probability for the mtDNA markers (Figure 2). Clade I is composed of only the NC/CG haplotypes, clade II includes the NAG, SAG and DRG haplotypes, and clade III is mainly composed by the SAG haplotypes and a few individuals from the NAG (Figure 2). The NC/CG is the sister group of clades II and III. The DRG samples compose a clade with a high posterior probability that is nested within clade II. Four individuals from the NAG, three individuals from Cuba and one from the DRG did not cluster within their regional groups (Figure 2), and these individuals were considered aliens, i.e., resulting from dispersal mediated by humans, as discussed in our previous work [32].

The tree topology indicates island colonization from mainland populations. The phylogenetic relationship recovered for the Dominican Republic and Cuban samples and the high Φ_ST_ between the DRG and NC/CG indicate that the populations from these two islands were derived from two different founder events. Cuba was colonized from North/Central America populations, and Dominican Republic was colonized from South American populations. Haplotypes from Jamaica and Trinidad and Tobago are equal or closely related to the haplotypes found in the North Amazon region, and these *C. hominivorax* populations were probably founded by the South American populations.

The SAG and NAG samples are distributed into two clades (Figure 2, Clade II and Clade III), but they do not form reciprocal monophyletic groups that are correlated with geographic distribution. Nevertheless, the samples from each regional group tend to cluster together into each main clade. Different hypotheses can explain the pattern observed for NAG and SAG; for example, (a) the SAG populations were derived from different colonization events from NAG or vice versa, (b) a population admixture event may have occurred between these groups, or (c) populations may have diverged recently, but because a sufficient period of time has elapsed, these new populations no longer share haplotypes. These hypotheses were tested in the ABC analyses (see below).

The Bayesian Skyline Plots revealed a complex demographic history for *C. hominivorax* populations (Figure 2; BSP graphs). Clades II and III present a population expiation that begun before the LGM, whereas clade II started expansion before clade III. However, the stronger signal of population expansion comes from the SAG populations, while NAG showed a more stable population size through time.

### Habitat suitability modeling

**Figure 3:**
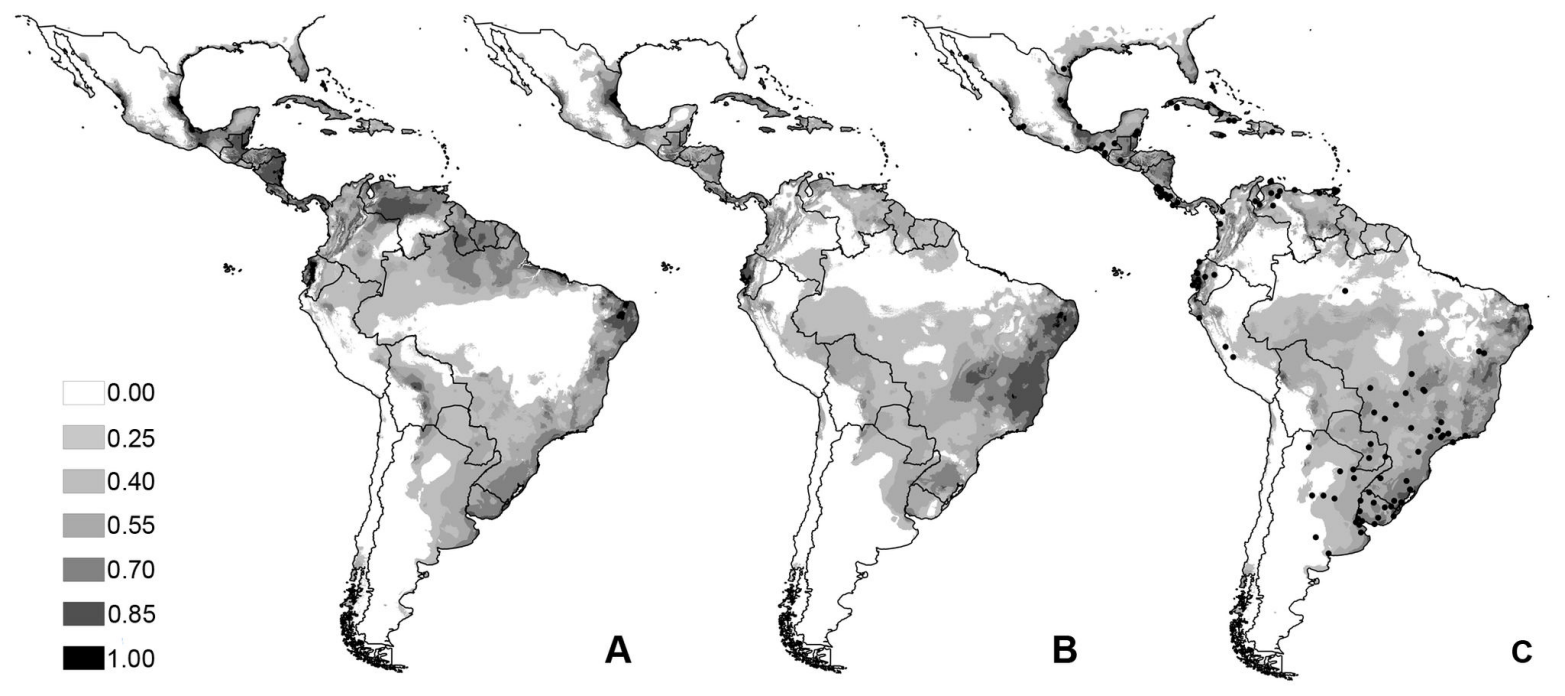
Maxent models for the potential distribution of *C. hominivorax*. A: Last Inter Glacial (LIG) conditions; B: The average Last Glacial Maximum (LGM) conditions for the CCSM and MIROC models; C: Present conditions (black points are geo-referenced sampling locations used for modeling). Maps are plotted with threshold of 0.25 in all of the analyses.

The habitat suitability models for *C. hominivorax* generated by Maxent are shown in Figure 3 (Figure 3A: LIG; 3B: LGM; and 3C: present). The AUC for the present model was 0.816 (SD = 0.05), 0.823 (SD = 0.060) for the MIROC-LGM, 0.823 (SD = 0.031) for the CCSM-LGM, and 0.822 (SD = 0.067) for the LIG, indicating that the outputs of our models are of high quality [40,48,62]. The models shows suitability areas at all times across the American continents, and an increase of the suitability areas in South America from the past to present. Models also show large suitable areas for *C. hominivorax* on both sides of the Amazon, in Central America and the Caribbean islands. All of the models also indicate that the Andes, the Amazon and the southern part of the continent were not suitable areas. The average model of LGM suggests possible connections between the North and South Amazon regions through the Andes, crossing the mountains in the south of Colombia and Peru.

### Approximate Bayesian computation

Evolutionary hypotheses to test population divergence without admixture revealed high posterior probabilities for the first event of population divergence occurring between North/Central America (NC) and South America and a second split between populations distributed in the North and South Amazon regions (the NAG and SAG, respectively) (Figure 1A; Sc1, Sc2, Sc3; Table 4). Among these, the Sc1 scenario describes a single expansion event that has the highest posterior probability. The tests that consider admixture revealed a high posterior probability for the Sc7 scenario, with an admixture event between the NAG and SAG after the split of the NC populations (Figure 1B; Table 4).

**Table 4:** ABC comparison between the hypothesized evolutionary scenarios.

Hypotheses	Probability of scenario (logistic regression)	Confidence interval (95%)
Population divergence		
**Sc1**	**0.4266**	**[0.3902-0.4629]**
Sc2	0.3159	[0.2836-0.3482]
Sc3	0.2558	[0.2265-0.2851]
Sc4	0.0007	[0.0005-0.0009]
Sc5	0.0007	[0.0005-0.0009]
Sc6	0.0003	[0.0002-0.0004]
Population admixture		
**Sc7**	**0.8600**	**[0.8172-0.9028]**
Sc8	0.1400	[0.0972-0.1828]
Best scenarios		
**Sc1**	**0.8251**	**[0.8019-0.8483]**
Sc7	0.1749	[0.1517-0.1981]

In the comparison between scenarios Sc1 and Sc7, Sc1 presented the highest posterior probability (*P* = 0.8251, 95% CI [0.8019, 0.8483], Table 4 and Figure 1A, 1B). For this scenario, none of the observed summary statistics deviate significantly from the simulated distributions (P > 0.05) according to model checking computation analysis.

The parameter estimates for the Sc1 scenario are given in Table 5 (graphical output are in Figure S1). A population divergence between North and Central America and South America occurred approximately 15,300-19,000 YBP, just after the LGM. The divergence between the South American regional groups (NAG and SAG) occurred approximately 9,100-11,000 YBP in the transition between the Pleistocene and Holocene, with a nearly coincident expansion of populations (7,200-8,200 YBP). The current population effective sizes for the three regional groups are much higher than the ancient population sizes, where N_SAG_ ~100 times, N_NAG_ ~20 times, and N_NC_ ~4 times higher than N_anc_.

**Table 5:** Parameter estimates for the best population divergence scenario (Sc1; table 3).

**Parameter**	**mean**	**median**	**mode**	**q025**	**q975**
N_anc_ (10^3^)	26.1	23.8	4.9	1.8	57.8
N_SAG_ (10^3^)	2420	2390	2080	1340	3500
N_NAG_ (10^3^)	542	549	575	279	775
N_NC_ (10^3^)	99	100	98	49	146
t_exp_ (generations x10^3^)	125	122	110	72	200
t_exp_ (years)	8.2	8.0	7.2	4.7	13.1
t_2_ (generations x10^3^)	167	159	139	79	300
t_2_ (thousand years)	11.0	10.4	9.1	5.2	19.7
t_3_ (generations x10^3^)	290	274	233	151	520
t_3_ (thousand years)	19.0	18.0	15.3	9.9	34.1
Mµ (10^-8^)	3.09	2.47	1.47	0.83	8.8

Parameter definitions are in Table 1.

## Discussion

Using the ABC analysis, we found that *C. hominivorax* populations diverged from north to south in the Americas. According to the best-supported scenario (i.e. Sc1), a first population split occurred between North/Central America and South America after the Last Glacial Maximum. The second population split occurred in the transition between the Pleistocene and Holocene and led to the formation of the regional groups in north and south of the Amazon region.

Phylogeographic breaks occurring between Central and South America and/or the North and the South Amazon regions are frequently described in the literature and have revealed long histories of isolation and divergence, with continued and ancient events affecting diversification across lineages. Some studies suggest that ancient events dating back to the Neogene and late Pleistocene were critical in shaping the Mesoamerican lineages [63-70]. Most of these works found that geographical barriers and/or the creation of new ecosystems explain the documented patterns of biotic evolution. Volcanic activity and isolation due to Pleistocene climate oscillations have also been suggested for the more recent divergences (e.g. [71]). In the Amazon, phylogeographic breakups have been documented for different taxa [5,8,72,73]. The South American biota have a complex evolutionary history mostly caused by the Andean uplift, the drainage shift of the entire Amazon basin during the Miocene, or periodic climate and landscape changes that took place in the Pliocene and Pleistocene [8,10,73-75]. Silva & Bates [9] have suggested that the existence of corridors connecting the South American open formations during the LGM might explain the current geographic distribution of taxa inhabiting open areas on both sides of the Amazon basin.

The divergences among *C. hominivorax* populations occurred during two climate periods, the first just after the LGM and the second at the transition between the Pleistocene and the Holocene. In addiction, the BSP shows a non-synchronous expansion of samples from SAG group in clade II and clade III. These results suggest that climate oscillations alone do not explain the genetic divergence between the North/Central American-South American lineages of *C. hominivorax* and the north Amazon-south Amazon lineages, but certainly could partially explain the observed pattern. The average of LGM habitat models show an area of medium to low suitability between North/Central America and South America, that could have been a climate barrier. All of the models show areas of low suitability for *C. hominivorax* in the Amazon region, suggesting that this region cannot be adequately occupied by the species. This result is consistent with the observations that *C. hominivorax* preferentially inhabits areas at the forest edge, where adults feed, rest and mate, and grazing areas, where females seek hosts to lay their eggs [76-79]. Even though, this species appears to have crossed this Amazon area in the warming period, when forests might have expanded [4,80]; thus the hypothesized corridors suggested by Silva and Bates [9] do not seem to explain the observed distribution pattern.

Alternatively, the association of the species with their hosts and its distribution, movement, and population expansion might explain this apparent paradox, and might have contributed to shape the observed phylogeographic pattern.

Human migrations have been linked to the introduction and spread of various human and livestock parasites to different habitats and hosts [81,82]. Although *C. hominivorax* can attack different hosts, we found a phylogeographic pattern coincident with the general pattern of human Native American populations spread throughout the continent [83-85].

Although the number, route and timing of human migration events into the Americas remain under intense debate [83-91], some general patterns have begun to emerge. Human Native American populations seem to have experienced a strong growth that started approximately 18,000 YBP (see 88). This population growth was followed by a “fast” spread (~2,000 years) along the Americas from a starting point in the North [84,92-94].

Our analyses indicated that *C. hominivorax* experienced population expansion during its dispersal towards its current range. Both ABC and BSP analyses show that the effective population size in the SAG group are bigger, suggesting that the species has found a favorable environment to reproduce and expand after crossing the Amazon region (e.g. different hosts). With ABC analysis, we estimated that the expansion occurred in the beginning of the Holocene. The BSP analyses show different patterns and times of population expansion for each regional group. The disagreement between the estimated times may be due to the incorporation of structured populations in the BSP, which might have biased the analyses (see 88), or because the ABC models tested here did not consider continuous population growth. However, the BSP shows that SAG retained a stronger signal of population expansion and it predates divergence. A likely explanation for the results is also a phenomenon called “surfing” [95,96], in which an allele could increase in frequency and be propagated by a wave of range expansion. In *C. hominivorax*, the expansion might have started in the north, but only SAG retained alleles that were present in the wave of south colonization, due to the high effective population size. Thus, results of the BSP complements ABC analysis and are very concordant with the North to South divergence pattern found in this work.

The phylogenetic analyses indicated that the colonization of the Caribbean islands by *C. hominivorax* took place after the first mainland split and originated from different source-populations, i.e., Cuba from North and Central America and the Dominican Republic from South America. Interestingly, Bodner et al. [84] found that human populations coming from South America colonized the Dominican Republic, and Mendizabal et al. [97] suggested that both North and South America contributed to the ancient human gene pool in Cuba. These data also suggest that humans might have played a central role in the spread of *C. hominivorax* throughout the Caribbean. This colonization scenario explains the high genetic variability and divergence between Caribbean island populations, as suggested in previous studies [30-32].

In our ABC analyses we avoid to explicit test more complex scenarios because our mitochondrial data constitute one-locus datasets and have little power to correctly rank asymmetrical models [98]. But other scenarios that couples for example independent colonization events and population expansion (see 99) might better explain the high genetic variability found in the *C. hominivorax* populations in the South Amazon region.

The data and sampling used in this work allowed us to determine the order and time of the divergence events, as well discuss the possible role of human migration in shaping the historical demography of *C. hominivorax*. This work provided us with an evolutionary framework that can support studies on a finer scale that are directed at understanding the genetic structure of *C. hominivorax* populations within regional groups.

## Supporting Information

Figure S1 **Approximate Bayesian Computation graphical output.**
(PDF)

Table S1 ***Cochliomyia hominivorax* sample locations, sizes and haplotype distributions.**
(XLS)

Table S2 ***Cochliomyia hominivorax* locations used in the habitat suitability modeling.**
(XLS)

Table S3 **Haplotype codes for each independent mtDNA fragment, combined haplotypes and Genbank accession numbers.**
(XLS)
