# Supplementary material for: The Phylogeographic History of the New World Screwworm Fly, Inferred by Approximate Bayesian Computation Analysis
Source: PLoS One. 2013 Oct 2;8(10):e76168. doi: 10.1371/journal.pone.0076168 (PMC3788763; doi:10.1371/journal.pone.0076168)
Supplement: Figure S1 — Approximate Bayesian Computation graphical output. (PDF) [file pone.0076168.s001.pdf]

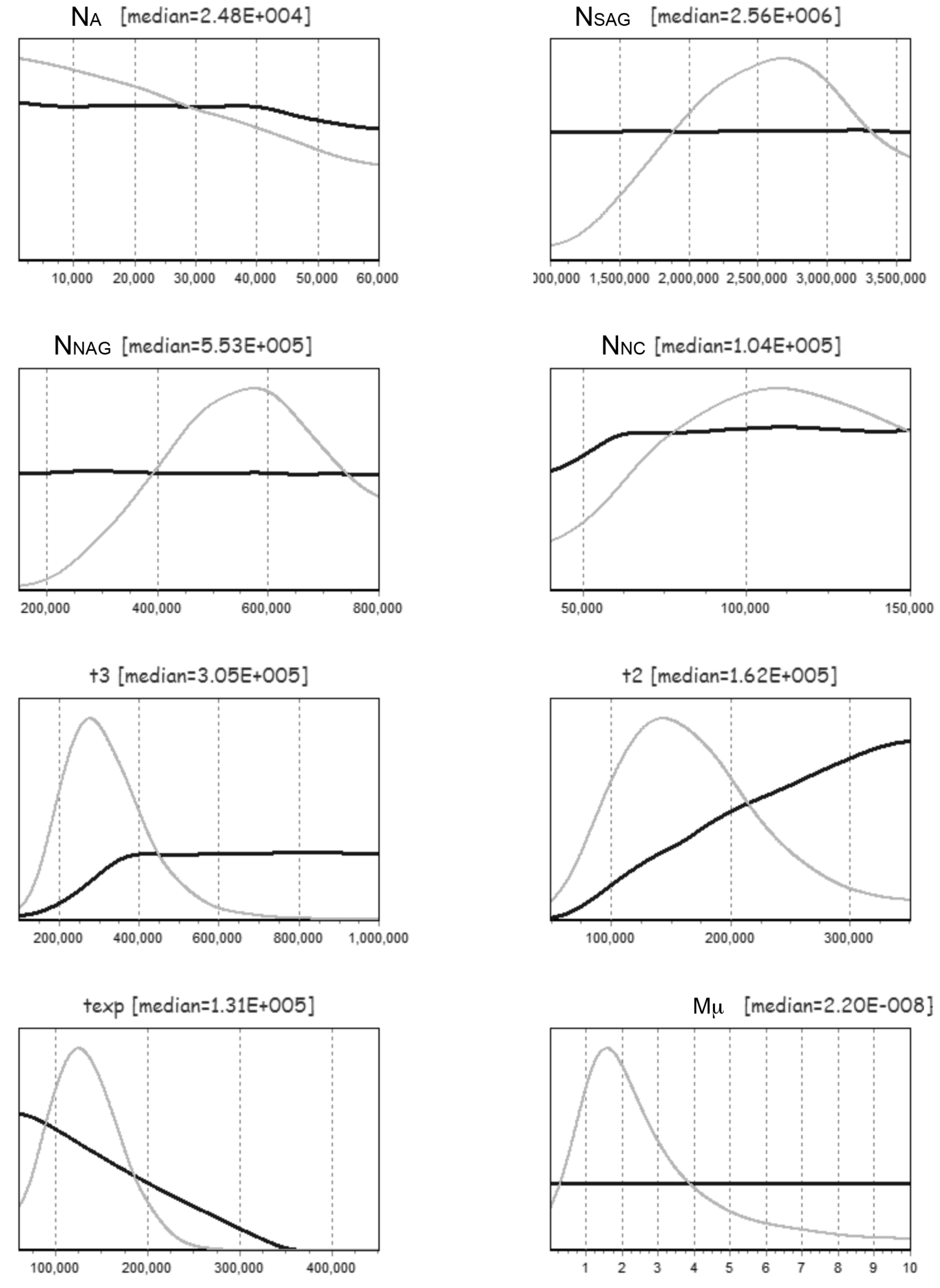

Figure S1. Approximate Bayesian Computation graphical output. It shows prior (in black) and posterior (in gray) distributions of original and composite parameters (estimation under scenario Sc1). Parameters definitions are in Table 1.
